# Secular trends and regional variations in pharmacotherapy of attention-deficit/hyperactivity disorder (ADHD) among children and adolescents in Germany

**DOI:** 10.1186/s12888-021-03409-6

**Published:** 2021-08-14

**Authors:** Manas K. Akmatov, Jakob Holstiege, Jörg Bätzing

**Affiliations:** Department of Epidemiology and Health Care Atlas, Central Research Institute of Ambulatory Health Care in Germany, Salzufer 8, 10587 Berlin, Germany

**Keywords:** ADHD, Claims data, Germany, Methylphenidate, Pharmacotherapy, Regional variations, Trends

## Abstract

**Background:**

The study aim was to examine the secular trends and regional variations in pharmacotherapy of children and adolescents with attention-deficit/hyperactivity disorder (ADHD) in Germany.

**Methods:**

We used nationwide drug prescription data of outpatient care (2009 to 2016). The study population comprised patients aged between 5 and 14 years with the diagnoses “hyperkinetic disorders” (ICD-10 code F90) (e.g. *n* = 262,766 in 2016). The examined drugs were methylphenidate, amphetamines, atomoxetine and guanfacine.

**Results:**

Overall, the proportion of patients received any prescription showed a decreasing trend over years (2010, 51%; 2016, 44%). The proportion of methylphenidate prescription was higher in Western than Eastern federal states. However, atomoxetine was more often prescribed in Eastern than Western federal states. The proportion of methylphenidate prescriptions issued by pediatric psychiatrists increased from 28% (2009) to 41% (2016).

**Conclusion:**

A decreasing trend in use of pharmacotherapy may be explained by prescription restrictions issued by the Federal Joint Committee in recent years.

## Background

Attention-deficit/hyperactivity disorders (ADHD) is one of the frequent neurodevelopmental disorders among children and adolescents. A recent population-based study in Germany showed a stagnating trend in the period between 2009 and 2016 [1]. In 2016, the prevalence amounted to 4.3% among children between 5 and 14 years [1]. The clinical guideline by the German Society for Child and Adolescent Psychiatry, Psychosomatics and Psychotherapy recommends as an initial step a non-pharmacological therapy, including psychosocial and psychotherapeutic interventions [2]. Accordingly, pharmacological intervention should only be initiated if a non-pharmacological therapy was unsuccessful. The first-line pharmacological treatment are stimulants (methylphenidate and amphetamines); the non-stimulants, atomoxetine and recently licensed guanfacine are second-line treatment options. Methylphenidate, available since 1954, is (by far) the most frequently prescribed stimulant for ADHD with atomoxetine being the second most frequently prescribed substance [3].

There has been controversy regarding ADHD over decades [4]. In particular, overdiagnosis and overmedication with stimulants were the subject of intense debate [5]. Previous studies reported increasing trends in prescription of both stimulants and non-stimulants in earlier years in Germany. Garbe et al. showed a dramatic increase in methylphenidate use in the period of 1990 to 2010; in this period prescription of methylphenidate increased by a factor 187 [6]. The prescription prevalence of methylphenidate on the population level doubled from 0.54% in 2000 to 1.1% in 2007 among children and adolescents [7]. Abbas et al. also observed a prevalence increase of both, methylphenidate and atomoxetine prescriptions from 2004 to 2012 [8]. The prescription prevalence of atomoxetine increased from 0% in 2004 to 2.1% in 2008 [8]. Due to an increasing trend in using stimulants the Federal Joint Committee (G-BA, “Gemeinsamer Bundesausschuss”) – the German regulatory agency for statutory health insurance – issued several prescription restrictions over the period of 2009 to 2014 [9]. In 2009 the stimulant prescription was restricted for preschool children. For school-aged children stimulants should only be prescribed as a second treatment option after a first-line nonpharmacological therapy without sufficient clinical improvement. In 2010 the G-BA extended the directives by restricting prescription of stimulants to certain specialist groups. There was a sign of stagnation or even a slight downward trend in prescription prevalence of methylphenidate between 2009 and 2014 [3]. Less is known about prescription patterns for other recently introduced stimulants, dexamfetamine and lisdexamfetamine and a non-stimulant guanfacine.

The aim of the study was to examine the secular trends and regional variations in pharmacotherapy of children and adolescents with ADHD. In particular, we were interested in the effect of restrictions issued by the G-BA on prescription prevalence and prescribing patterns.

## Methods

### Data

We used nationwide SHI-physician outpatient prescription data from the years 2009 to 2016. The data contain all prescriptions and diagnoses of statutory health insured individuals (SHI), who visited a SHI-authorized physician at least once per year. SHI-insurees account for about 87% of the total German population. In brief, prescriptions are issued by SHI-authorized physicians and redeemed by the patients in a pharmacy. Amongst other things, the data contain the date of prescription and dispensation, the anatomical therapeutic chemical (ATC) code, and the drugs’ generic and trade names. In the current analysis we considered the ATC codes from the group N06B exclusively used for ADHD therapy (“psychostimulants, agents used for ADHD and nootropics”). In Germany, the ATC classification is published annually by the German Institute of Medical Documentation and Information (DIMDI). The examined drugs were methylphenidate (ATC: N06BA04), atomoxetine (N06BA09), dexamfetamine (N06BA02), lisdexamfetamine (N06BA12) and guanfacine (N06BA14). Finally, data contain information on physicians (e.g. specialty) and patients (sex, age [in years] and region of residence). The latter consists of 17 regional Associations of Statutory Health Insurance Physicians (ASHIPs). Of them, 15 ASHIPs correspond to 15 German federal states whereas the federal state of North-Rhine-Westphalia has two ASHIPs. The data were used in accordance with Section 300 (2) of the Social Code V (“Sozialgesetzbuch V”).

### Study population

The study population comprised children and adolescents aged between 5 and 14 years diagnosed with ADHD (*n* = 274,202 in 2009; *n* = 262,766 in 2016). Diagnoses are coded according to the German modification of the 10th edition of the International Classification of Diseases and Related Health Problems (ICD-10-GM) [10]. We defined a child or adolescent having ADHD if the ICD-10 code F90 „hyperkinetic disorders “with an additional modifier “confirmed diagnosis” was coded in at least two quarters of a corresponding year [1].

### Statistical analysis

We calculated the annual prescription prevalence of stimulants and non-stimulants among children and adolescents with the diagnosis ‘ADHD’, total (at least one prescription of any medication) and separately for each medication for the period of 2009 to 2016. The curves of prevalence over time were fitted using a cubic spline function. The joinpoint regression analysis was then used to assess time trends in prescription prevalence. This was performed for prevalence of at least one prescription and separately for each medication (dependent variables) and year (independent variable) with the Joinpoint Trend Analysis Software (version 4.9.0.0) available from the National Cancer Institute [11].

In addition, we performed univariate cross-sectional analysis by sex, age and region (2016). Analyses were performed with the R Foundation for Statistical Computing, version 3.4.4 (https://www.r-project.org).

## Results

### Description of the study population

The size of the study populations (i.e. children and adolescents between 5 and 14 years diagnosed with ADHD) ranged across years between 262,000 and 287,000 (Table 1). The proportion of boys than girls was higher in all years with nearly three quarters of boys in 2016. Around 25% of all patients were in the age groups, 9–10, 11–12 and 13–14 years, respectively.

**Table 1:** Demographic characteristics of the study populations

Characteristics	2009	2010	2010	2012	2013	2014	2015	2016
Patients (n)	274,202	283,224	287,155	278,234	270,348	273,276	265,922	262,766
Sex (%)								
boys	69.5	69.6	69.5	70.9	73.0	73.3	74.3	74.3
girls	20.1	20.7	21.1	21.7	22.5	23.2	23.9	24.1
unknown	10.3	9.7	9.4	7.3	4.5	3.5	1.8	1.5
Age groups (%)								
5–6	6.5	6.5	6.1	5.9	5.8	6.7	6.6	6.5
7–8	17.7	17.1	16.7	16.4	16.1	16.3	16.8	17.2
9–10	27.4	26.9	26.1	25.7	25.4	25.3	25.2	25.4
11–12	26.9	26.9	27.4	27.5	27.1	26.5	26.5	26.4
13–14	21.5	22.6	23.6	24.5	25.5	25.2	24.9	24.5

### Prescription prevalence

**Fig. 1:**
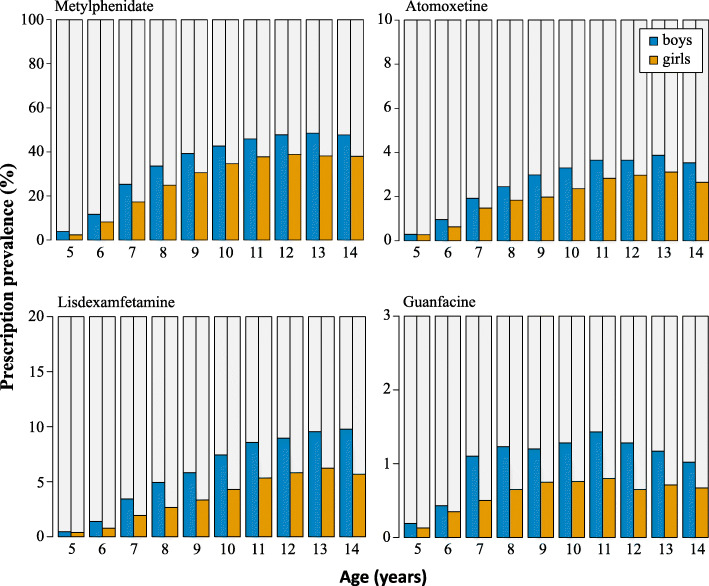
Prevalence of at least one prescription of methylphenidate, atomoxetine, lisdexamfetamine and guanfacine among children and adolescents with ADHD in 2016, by sex and age

Of the 262,766 children and adolescents with ADHD in 2016, 116,021 (44%) received at least one prescription of (any) stimulant or non-stimulant. The most frequently prescribed medication was methylphenidate; 38% of all children and adolescents with ADHD received this prescription, followed by lisdexamfetamine (6.5%), atomoxetine (2.9%) and guanfacine (1.1%). The prevalence of dexamfetamine prescriptions was negligible (0.69%). The prescription prevalence of all medications was higher among boys than girls in all age groups (Fig. 1). In addition, the prescription prevalence showed a strong age-dependence; for example, the prevalence of methylphenidate prescriptions was the lowest among the youngest children and increased linearly until the age of 12 years in both, boys and girls. A similar pattern of association was observed for all other stimulants and non-stimulants (Fig. 1, data for dexamfetamine not shown).

### Secular trends

**Fig. 2:**
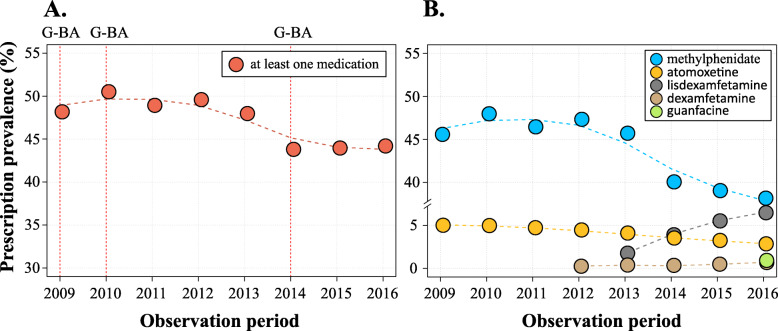
Prevalence of at least one prescription of any (**A**) and specific medication (**B**) in the years 2009 to 2016. The curves were fitted using a cubic spline function. Panel A: vertical lines refer to the introduction of prescription restrictions issued by the Federal Joint Committee (G-BA) in the respective years. Panel B: Dexamfetamine, lisdexamfetamine and guanfacine were licensed in the years 2012, 2013 and 2016 in Germany, respectively

The trends in prescription prevalence over the period of 2009 to 2016 are presented in Fig. 2. The highest prescription prevalence of at least one (any) medication was observed in 2010; nearly every second child with ADHD received a prescription (Fig. 2A). The prescription prevalence displayed a downward trend and reached 44% in 2016 (annual percent change, APC = -2%, *p* = 0.011). Figure 2B shows trends in prevalence for each medication. The prevalence of methylphenidate prescriptions decreased over time from 46% in 2009 to 38% in 2016; the trend was nonlinear with a pronounced drop of prescription prevalence in 2014 (APC = -5.8%, *p* = 0.047). The prevalence of atomoxetine prescriptions also showed a downward trend with one joinpoint (APC from 2009 to 2012, − 4.4%, *p* = 0.025 and from 2012 to 2016, − 10.4%, *p* = 0.001). Overall, in 2009 and 2016 its prevalence was 5.0 and 2.9%, respectively, corresponding to a relative decrease of 42%. The prevalence of lisdexamfetamine prescriptions increased from 1.7% in 2013 (i.e. the year of license) to 6.5% in 2016 (APC = + 44%, *p* = 0.078). The prescription prevalence of dexamfetamine (which was licensed in 2012 in Germany) displayed a rising trend (APC = + 26.6%, *p* = 0.026), but was very low in all years (2012, 0.26%; 2016, 0.69%).

### Regional variations

**Fig. 3:**
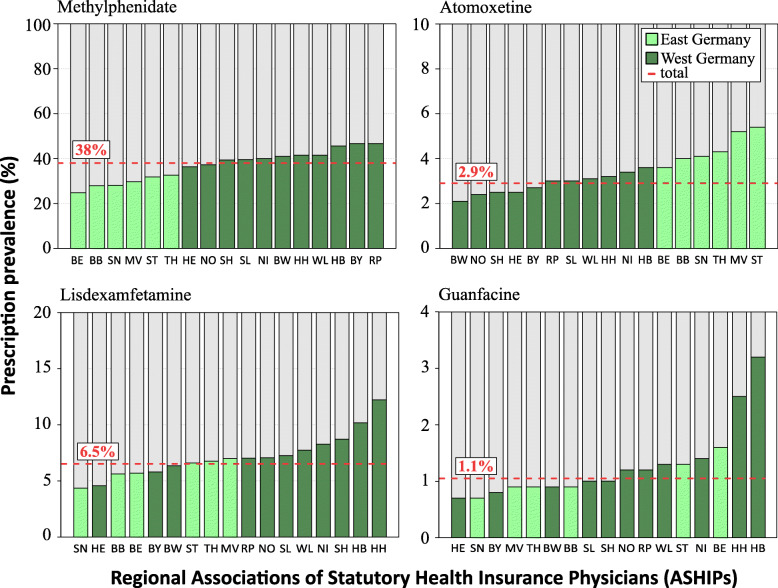
Prevalence of at least one prescription of methylphenidate, atomoxetine, lisdexamfetamine and guanfacine among children and adolescents with ADHD in 2016, by region. East and West Germany refer to the former German Democratic Republic (GDR) and Federal Republic of Germany (FRG) before the reunification of both states in 1990, respectively. BW, Baden-Württemberg; BY, Bavaria; BE, Berlin; BB, Brandenburg; HB, Bremen; HH, Hamburg; HE, Hessen; MV, Mecklenburg-Western Pomerania; NI, Lower Saxony; NO, North Rhine; WL, Westphalia-Lippe; RP, Rhineland-Palatinate; SL, Saarland; SN, Saxony; ST, Saxony-Anhalt; SH, Schleswig-Holstein; TH, Thuringia

Regional variations in prescription prevalence of stimulants and non-stimulants in 2016 are presented in Fig. 3. The prescription of methylphenidate and atomoxetine showed a clear distinctive regional pattern. The prescription prevalence of methylphenidate was lowest in all Eastern federal states and Berlin (between 25 and 33%) and highest in all Western federal states with the highest values in Bremen (46%), Bavaria (47%) and Rhineland-Palatinate (47%). A completely different regional pattern was observed for atomoxetine prescriptions; the prevalence was higher in Berlin and all Eastern than in Western federal states (Fig. 3). We observed no clear difference between East and West for other medications. Of note, the prescription prevalence of the lately licensed lisdexamfetamine (2013) and guanfacine (2016) was much higher in the federal states of Bremen and Hamburg as compared to other states (Fig. 3).

### Methylphenidate prescription by physician groups

**Fig. 4:**
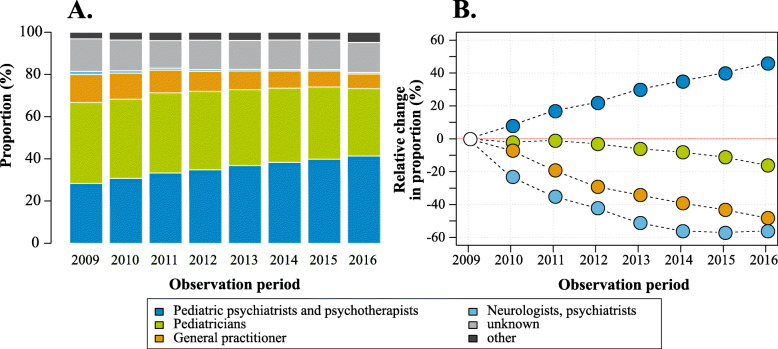
Proportions of physician groups prescribing methylphenidate in the years 2009 to 2016 (**A**) and its relative change over years (**B**)

In its second directive in 2010 the G-BA issued explicitly the physician specialties to prescribe stimulants. The stimulants were only allowed to be prescribed by specialist physicians including pediatric psychiatrists, specialists for behavioral disorders in children and adolescents etc. Only in exceptional cases stimulants could be prescribed by pediatricians and general practitioners (GPs). We examined physician groups who prescribed the most frequent stimulant – methylphenidate – in 2009 and how it changed over the observation period (Fig. 4A). In 2009, 38% of children and adolescents with ADHD received methylphenidate prescription from pediatricians. The second biggest physician group prescribing methylphenidate were pediatric psychiatrists and psychotherapists (28%). About 13% of all children with ADHD received a prescription from GPs. This pattern changed over time (Fig. 4A and B). The proportion of prescribing pediatric psychiatrists and psychotherapists increased constantly and reached 41% in 2016. In contrast, the role of other specialist groups including pediatricians and GPs decreased.

## Discussion

Using the nationwide prescription data we examined the trends and regional variations in pharmacotherapy of ADHD among children and adolescents in Germany. Overall, we observed a decreasing trend in prescription prevalence of stimulants and non-stimulants over the period 2009 to 2016. In addition, there were regional variations in prescription.

Previous research in Germany observed rising trends in use of stimulants [12]. The G-BA responded to these trends in using pharmacological treatment in Germany by issuing several prescription restrictions of stimulants [9]. The first restrictions were introduced in 2009 and extended in 2010. The aim of these directives was to avoid overuse of pharmacological therapy, in particular among those children not needing pharmacological treatment (e.g. preschool children and children with a possible false positive diagnosis). Indeed, we observed an age-related association in prescriptions of all substances and a downward trend for methylphenidate, the most frequently prescribed stimulant and atomoxetine. In terms of age, the proportion of younger children (in particular preschool children) with a prescription was very low, which in addition to the G-BA restrictions can also be explained by the clinical guideline of the German Society for Child and Adolescent Psychiatry, Psychosomatics and Psychotherapy, which recommends first a non-pharmacological therapy, including psychosocial and psychotherapeutic interventions in children younger than 6 years and a pharmacotherapy in children between 3 and 6 years in exceptional cases. In terms of time trends we observed a slight decline in methylphenidate prescriptions in the period of 2009 to 2013 and a pronounced drop in 2014 with a subsequent decline thereafter. Atomoxetine, a non-stimulant, also displayed a downward trend. Bachmann et al. observed a stagnation in total ADHD medication use among children and adolescents younger than 19 years between 2008 and 2012 [13]. In another study, Bachmann et al. reported first evidence of a decline in medication use among children and adolescents by comparing prescription prevalence in 2009 and 2014 [3]. Both studies did not differentiate across various substances. Finally, a recently published study reported similar trends in medication use among children (≤16 years) in a 10-year period of 2008 to 2018 [14]. This study was based on a relatively small sample of children and adolescents (*n* = 33,192). All five substances were included in the study, however, only overall trends for ADHD medication were reported without differentiation of specific substances.

We observed the only increase in prescription prevalence of amphetamines. In 2013 licenced lisdexamfetamine showed a nearly 4-fold increase within the first 3 years (2013, 1.7%; 2016, 6.5%). In the year of license lisdexamfetamine was the third most frequently prescribed medication. In 2014 it was already the second most frequently prescribed medication. Dexamfetamine prescriptions increased by a factor 2.6 from 2012 (0.26%) to 2016 (0.69%). However, overall, the proportion of children receiving amphetamine prescriptions was low. Of note, the introduction of new medications and an increase of amphetamine prescriptions did not result in an overall increase of children prescribed medications.

One possible explanation for declining prescription trends would be the decrease in prevalence of ADHD. However, in the same period of time we did not observe a decrease in prevalence of ADHD [1]. The prevalence of ADHD in the same study population of 5 to 14 years old children and adolescents showed a stagnating trend between 4.1% in 2009 and 4.3% in 2016 [1]. In conclusion, the declining trends may be explained by the above-mentioned restrictions issued by G-BA.

Regionally we observed distinct patterns, which have not been described previously. Methylphenidate was more commonly prescribed in Western than in Eastern federal states. A similar finding was seen for lisdexamfetamine although the regional pattern was not so clear. On the contrary, atomoxetine prescriptions were more common in Eastern federal states. The reasons for these variations are not known and need to be explored. Grobe et al. examined regional differences in methylphenidate use on a district level and observed a high correlation between ADHD prevalence and medication use (r = 0.84) [15]. We did not observe such a correlation on the level of federal states (Spearman’s rho = 0.046, *p* = 0.86). Unfortunately, a regional analysis on a district level is not possible as this information is not available in the dataset due to the current data protection regulations. One possible explanation for regional variations in prescription prevalence is the availability of various physician groups. For instance, the density of pediatric psychiatrists and psychotherapists is higher in Western than Eastern federal states [16]. This is also supported by the fact that the prescription prevalence of the lately introduced amphetamines, lisdexamfetamine and dexamfetamine, was highest in both Hanseatic cities Bremen and Hamburg. Both cities have a high density of pediatric psychiatrists and psychotherapists as compared to other regions.

We observed a shift towards increasing proportion of physician groups specialized in psychiatry and psychotherapy prescribing stimulants. Whereas in 2009 the most frequent physician group prescribing methylphenidate were pediatricians, this pattern changed gradually over years. In the last year of observation (2016), the most frequent physician group prescribing methylphenidate were pediatric psychiatrists and psychotherapists. The proportion of pediatricians showed a slow decrease over years. However, in 2016 every third patient with ADHD still received a prescription from a pediatrician. The proportion of GPs prescribing methylphenidate showed a more rapid decline; e.g. in 2016 only every 15th patient with ADHD received a prescription from this physician group. Overall, we observed changes in prescribing patterns which can be attributed to the second directive of the G-BA issued in 2010. The G-BA restricted prescriptions to certain physician groups. The prescription of stimulants by pediatricians and GPs was only allowed in exceptional cases.

### Strengths and limitations

We used the nationwide outpatient drug prescription data of all statutory health insured individuals covering 87% of the total German population. The dataset does not contain information on individuals insured privately. The latter have a higher socio-economic status, which was associated with a lower risk of ADHD [17]. We cannot rule out possible differences in use of pharmacological treatment between statutory and privately insured children. On the other side, the dataset may yield the complete picture of stimulant and non-stimulant prescriptions as nearly 99% of all ADHD diagnoses (and pharmacological treatment) occur in outpatient care [15]. Furthermore, SHI-physicians code diagnoses according to the ICD-10. We used the code F90 “hyperkinetic disorders” to identify children and adolescents with ADHD. Since there are differences in diagnostic criteria based on the ICD-10 – which codes hyperkinetic disorders – and the Diagnostic and Statistical Manual of Mental Disorders (DSM) – which codes ADHD – the risk of misclassification in our study may not be ruled out.

## Conclusions

The prescription prevalence of stimulants and non-stimulants showed a decreasing trend over the period of 2009 and 2016 among children and adolescents. In addition, the proportion of pediatric psychiatrists prescribing stimulants increased whereas the proportion of prescriptions issued by pediatricians and GPs decreased. This might be the effect of strict directives of the G-BA issued in recent years. These directives may contribute to increasing awareness among physicians regarding the need of a pharmacological therapy of ADHD.

## Data Availability

The datasets analysed during the current study are not publicly available due to data protection regulations by the Code of Social Law (Sozialgesetzbuch, SGB V).

## Abbreviations

ADHD: attention-deficit/hyperactivity disorders
APC: Annual percent change
ASHIPs: Regional associations of statutory health insurance physicians
ATC: Anatomical therapeutic chemical
DIMDI: German institute of medical documentation and information
DSM: Diagnostic and statistical manual of mental disorders
G-BA: Gemeinsamer bundesausschuss (federal joint committee)
GP: General practitioners
ICD-10: International classification of diseases and related health problems, 10th edition
SHI: Statutory health insurance
